# Mental automatism and exogenous psychosis: the originality of De Clérambault’s and Karl Bonhoeffer’s theories in substance-related psychoses

**DOI:** 10.3389/fpsyt.2025.1532730

**Published:** 2025-01-30

**Authors:** Valerio Ricci, Alessandro Sarni, Giuseppe Maina

**Affiliations:** ^1^ San Luigi Gonzaga Hospital, University of Turin, Orbassano, Italy; ^2^ Department of Neurosciences “Rita Levi Montalcini”, University of Turin, Turin, Italy

**Keywords:** substance-induced psychosis (SIP), schizophrenia, mental automatism, exogenous psychosis, Gaëtan de Clérambault, Karl Bonhoeffer, cannabis, psychostimulants

## Introduction

The relationship between substance use and the onset of psychosis has been a subject of clinical and academic debate for centuries. The potential for cannabis to trigger psychosis was first suggested by Grose in 1772, who documented issues related to cannabis use in India (1). Karl Jaspers later explored this connection in his Allgemeine Psychopathologie (1959), describing toxic intoxications as “model psychoses” that mimic acute episodes, noting their intermediate processes remain unclear (2). Before Jaspers, Jacques-Joseph Moreau de Tours, in the 19th century, argued for a continuum between normal and pathological mental states based on his observations of hashish users. He proposed that delusions seen in mental illness and hashish intoxication share a psychological nature akin to dreams (3). The 1960s and 1970s brought increased cannabis use and heightened interest in its potential to induce acute psychosis and chronic schizophrenia (4, 5). More recently, the availability of novel psychoactive substances (NPSs) and high-potency drugs like cannabis and cocaine has raised global concerns, especially among adolescents and young adults—groups particularly susceptible due to developmental and behavioral factors (6–8). This trend has been linked to an increase in psychotic episodes, underscoring the urgency of understanding these disorders’ mechanisms (9–12). Substance-induced psychotic disorder (SIP), as classified in the DSM-5, is marked by delusions and/or hallucinations occurring during or shortly after substance use or withdrawal (13). Clinically, SIP presents with sudden symptoms such as paranoia, agitation, and hallucinations, with severity influenced by the substance, dosage, and individual susceptibility. Psychostimulants like cocaine and methamphetamine provoke intense psychotic episodes, while hallucinogens like LSD cause perceptual and cognitive distortions, often with vivid hallucinations (14, 15). Chronic substance use may lead to lasting brain changes. Neuroimaging studies link prolonged use of cannabis, cocaine, and methamphetamine to structural and functional alterations in regions essential for cognition, emotional regulation, and reward processing, contributing to enduring deficits (16–19). Repeated exposure to potent substances, particularly synthetic cannabinoids, is associated with prolonged psychosis and cognitive impairments. Risk factors for SIP include male gender, shorter durations of untreated psychosis, pronounced positive symptoms at onset, and lower academic achievement in adolescence (20–23).

This study examines the psychopathological foundations of SIP through the perspectives of Gaëtan de Clérambault and Karl Bonhoeffer. De Clérambault’s theory of “mental automatism” (24) emphasizes uncontrollable intrusions into consciousness, creating ego-dystonic experiences. Bonhoeffer’s concept of exogenous psychosis (25) highlights the interaction between neurobiological vulnerabilities and external triggers. These frameworks provide a complex understanding of SIP and inform strategies for early diagnosis and treatment.

## Subsection relevant for the subject

### De Clerambault and automatism in psychosis

Gaëtan Gatian de Clérambault (1872–1934), a leading early 20th-century psychiatrist, is renowned for his theory of mental automatism. Working at the Paris Prefecture of Police’s Special Infirmary for the Insane, he conducted over 13,000 psychiatric evaluations (24). In 1925, he formally defined mental automatism as a syndrome involving involuntary motor, sensory, and ideo-verbal phenomena. Patients experienced thoughts, sensations, or movements as foreign intrusions, disrupting their sense of agency (26). De Clérambault identified two types: positive automatism, involving intrusive additions like unbidden thoughts, and negative automatism, marked by suppressed mental processes. His work linked these phenomena to early psychosis, often preceding hallucinations (27, 28).

Within his framework, Gaëtan de Clérambault identified two subtypes of mental automatism: minor (petit automatisme) and major (grand automatisme). Minor automatism involved subtle, emotionally neutral intrusions that minimally affected functioning. In contrast, major automatism caused significant sensory and motor disruptions, leading to confusion, distress, and cognitive interference. Both minor and major automatisms are subtypes of positive mental automatism, characterized by intrusive mental additions such as unbidden thoughts, sensations, or actions. Progression from minor to major automatism often signaled a deepening detachment from one’s thoughts and actions, culminating in psychotic episodes.

De Clérambault argued that mental automatism, not individual symptoms like hallucinations, was the central mechanism of psychosis. He posited that psychosis arises from disrupted cognitive processes, not specific behaviors. Influenced by earlier French psychiatry, including Jules Baillarger and Jules Séglas, he unified intrusive experiences under a comprehensive framework, redefining psychosis as rooted in automatism rather than symptom clusters (23, 29).

De Clérambault viewed psychosis as a unified condition with mental automatism as its core process. He argued that automatic phenomena could lead to various delusional beliefs, shaped by how individuals interpreted intrusive experiences. For example, bodily sensations might result in hypochondriacal or persecutory delusions. This approach emphasized psychosis as a singular entity, with delusional content arising from cognitive interpretations of automatism. A firm organicist, de Clérambault believed mental automatism stemmed from biological disruptions, such as infections, intoxication, or brain lesions. He rejected psychological explanations, asserting that the consistent, mechanistic patterns of automatism indicated physical origins rather than unconscious conflicts (30).

De Clérambault’s work suggested that mental automatism was not only a key component of psychosis but also the primary driver of its symptomatology. He proposed that delusions were secondary responses of the rational mind to subconscious, intrusive phenomena. From his perspective, delusions served as attempts to explain strange sensations or ideas, with the mind attributing these experiences to external sources in order to make sense of the internal disturbance. In his description, a person affected by mental automatism might go through an *incubation period* during which the automatic phenomena would become more pervasive, gradually intruding upon and reshaping their broader mental life. This period of confusion often involved conflicting thoughts and sensations, which the individual attempted to rationalize, ultimately forming a delusional framework that incorporated these experiences (31, 32). A key aspect of de Clérambault’s theory was his emphasis on the non-sensory nature of mental automatism. He argued that these intrusive experiences were abstract disruptions of thought and consciousness, affecting cognition without necessarily forming delusional beliefs. These experiences may involve vague or fragmentary multisensory elements, but they remain fundamentally different from fully formed sensory hallucinations. He introduced the concept of “thought echo,” where thought processes duplicated over time, creating dislocation and disturbance without triggering immediate delusions. This distinction allowed de Clérambault to propose a broader definition of automatism, independent of sensory hallucinations or other psychotic symptoms (33).

### Bonhoffer and exogenous psychosis

De Clérambault’s work identifies a core dissociative mechanism that underpins involuntary phenomena, forming the foundation for delusions and sensory distortions like hallucinations. His insights link dissociation to psychopathological traits and renew interest in “exogenous” psychoses, contrasting them with the “endogenous” model that emphasized genetic inheritance and progressive degeneration. Karl Bonhoeffer (1868–1948) (25) revisits and refines this dichotomy, asserting that psychoses could stem from external factors and were often reversible. Drawing from his work in Breslau and Berlin, Bonhoeffer studied exogenous psychoses caused by alcohol and trauma, conditions overlooked in Kraepelin’s classification. He distinguished between degenerative-endogenous and reversible-exogenous psychoses, advocating for a broader understanding of mental illness shaped by both internal and external factors (34–36).

Bonhoeffer recognized the complexity of mental health, noting the frequent overlap of exogenous and endogenous causes, making clear distinctions challenging. He stated, “Pure forms of exogenous or endogenous origins are rare in practice … differentiation is challenging.” This perspective highlights the interplay of environmental stress, trauma, genetic predispositions, and neurobiological influences in psychiatric conditions. His approach advocates for moving beyond rigid categories to consider how these factors interact in the development of disorders. Bonhoeffer’s work emphasizes external pathogenic influences, such as substances, as triggers acting on underlying endogenous vulnerabilities. This concept underpins the notion of “lysergic psychoma,” introduced by Cargnello and Callieri (1963) (37, 38), based on Bonhoeffer’s exogenous model. It describes a syndrome involving alien intrusions into thought, sensory distortions, and hallucinations (15, 39). Bonhoeffer included lysergic psychosis within his model of hexogen psychosis, where an external noxious agent (noxa) directly instigates the onset of a psychotic episode. His initial studies were based on his work with alcohol-dependent patients in Breslau, under Wernicke’s guidance. Bonhoeffer’s goal was to uncover the root of a functional impairment—what he termed a hexogen complex—which, when combined with an existing endogenous predisposition, could lead to a full psychotic episode. His research focused on states of consciousness impairment, including symptoms ranging from disorientation and dream-like perceptions to delirium and stupor.

Bonhoeffer’s exogenous model, developed alongside Bleuler’s endogenous framework, offers insight into lysergic-induced psychosis as a distinct ego-dystonic experience (40–42). Individuals perceive “foreign thoughts” entering their minds, often accompanied by vivid visual and tactile hallucinations and delusional beliefs. While some self-awareness and control are retained, repeated episodes or prolonged drug use may weaken coping mechanisms, leading to persistent intrusions and deeper psychosis. Unlike typical psychotic delusions, drug-induced delusions involve temporary disruptions in spatial and temporal orientation. Maintaining coherence with shared reality is crucial to prevent escalation. This condition aligns with Bonhoeffer’s concept of exogenous psychosis, emphasizing dissociation, introspection, and temporary distortions, offering a framework to understand lysergic psychoma’s role in psychotic states (43).

## Discussion

At this point, we can define the significance of mental automatism as the psychopathological foundation of exogenous psychosis, particularly in the context of substance-induced psychotic disorders, from various perspectives.

The initial perspective is that De Clérambault’s research on mental automatism, conducted from 1920 to 1926, focused on the experience of uncontrollable and fragmented mental phenomena, anticipating what Schneider would later term “Gematch” (44). This view challenged existing psychosis theories by emphasizing organic and mechanistic roots. Moreover, De Clérambault developed the concept of mental automatism after observing numerous cases of intoxicated individuals exhibiting severe psychotic manifestations caused by substances like absinthe and alcohol, thus underscoring the biological foundations of the phenomenon. His observations illuminated the complex connections between neurobiological processes and psychopathological expressions. Additionally, De Clérambault identified a dissociative core at the root of secondary psychopathological symptoms. Building on this model, the theory of exogenous psychosis emerges: for instance, lysergic psychosis can be metaphorically understood as a “foreign body” (induced by the substance) that is perceived as such by a self that remains intact and self-aware.

The correlation of these two theories could broaden our understanding of the diverse manifestations of substance-induced psychosis, framing them as ego-dystonic experiences characterized by vivid hallucinations and cognitive disturbances. As a clinical example, frequent disturbances in body awareness (cenesthesia) and psychosensory experiences—particularly visual alterations—are key clinical symptoms of substance-induced psychosis, supported by the activation of mental automatism (45, 46). This phase typically begins in a ‘twilight state’ of consciousness, which can be metaphorically framed as a *chiaroscuro* state of mind, marked by intense visual hallucinations and significant somatosensory phenomena (47). As mental automatism progresses, and consequently the lysergic psychoma, continuous alterations in cognitive, sensory-perceptual, and motor domains emerge, eventually leading to a full-blown delusional state. These delusions, often paranoid, intertwine with the individual’s preexisting personality traits. Rather than appearing as revelations, these delusions act as confirmations of powerful sensory experiences. Over time, the psychotic experience becomes ego-syntonic, as continuous substance use and recurrent psychotic episodes gradually weaken critical self-awareness (48, 49). In a more structured clinical model, the initial symptoms in cases of toxic psychosis are primarily sensory-perceptual and are strongly linked to the prolonged use of hallucinogenic, dissociative, or stimulatory substances. During this state induced by these substances, individuals are particularly susceptible to hallucinatory misinterpretations, as a narrowed consciousness distorts perceptions. This often results in sensations of itching, irritation, pain, or burning, which the person might perceive as parasitic infestations. These sensations can be so overwhelming that they significantly alter the individual’s global bodily awareness (cenesthesia). The combined effect of sensory irritation and an altered state of consciousness creates a sense of bodily passivity associated with mental automatism; the individual remains aware, critical, and seeks assistance, yet feels powerless to control the progression of the psychosis (50) Analyzing the neural basis of mental automatism through neuroimaging tools like fMRI and PET scans highlights key brain regions, such as the prefrontal cortex and limbic system, involved in its pathophysiology (51–53). These areas, crucial for sensory processing and emotional regulation, are often disrupted by psychostimulants, initially causing subtle irritation that can escalate into full-blown psychosis (54).

In conclusion, the onset of psychiatric symptoms from potent substances is well-documented, often beginning with reversible psychotic episodes. Prolonged, high-dose use increases the risk of persistent disorders. De Clérambault’s theory of mental automatism and Bonhoeffer’s exogenous psychosis model offer critical insights. De Clérambault emphasized uncontrollable, alien phenomena central to psychosis, paralleling lysergic psychoma in substance-induced cases. Bonhoeffer highlighted how external agents interact with neurobiological vulnerabilities to trigger psychosis. Together, these frameworks suggest that prolonged exposure erodes the mind’s resilience, enabling chronic psychosis. Revisiting these theories enhances diagnostic approaches for exogenous psychoses and bridges understanding between endogenous and exogenous psychotic forms (55–57).

## References

[1] Grose JH. A Voyage to the East Indies Vol. 2. . London: S. Hooper (1772).

[2] Jaspers K. Allgemeine Psychopathologie. 1st ed. Berlin/Heidelberg, Germany: Springer (1913).

[3] Moreau JJ. Du Hachisch et de l’Aliénation Mentale . In: Études Psychologiques. De Fortin, Masson et Cie, Paris (1845). p. 30–1.

[4] Fekih-Romdhane F Alhuwailah A Shuwiekh HAM Stambouli M Hakiri A Cheour M. Development and initial validation of the cannabis-related psychosis risk literacy scale (CPRL): a multinational psychometric study. BMC Psychiatry. (2024) 24:298. doi: 10.1186/s12888-024-05727-x 38641784 PMC11027227

[5] van Os J Bak M Hanssen M Bijl RV de Graaf R Verdoux H. Cannabis use and psychosis: a longitudinal population-based study. Am J Epidemiol. (2002) 156:319–27. doi: 10.1093/aje/kwf043 12181101

[6] Bloomfield MA Ashok AH Volkow ND Howes OD. The effects of Δ9 tetrahydrocannabinol on the dopamine system. Nature. (2016) 539:369–77. 10.1038/nature20153 PMC5123717 27853201

[7] Farnia V Farshchian F Farshchian N Alikhani M Sadeghi Bahmani D Brand S. Comparisons of voxel-based morphometric brain volumes of individuals with methamphetamine-induced psychotic disorder and schizophrenia spectrum disorder and healthy controls. Neuropsychobiology. (2020) 79:170–8. doi: 10.1159/000504576 31794972

[8] McKetin R. Methamphetamine psychosis: insights from the past. Addiction. (2018) 113:1522–7. doi: 10.1111/add.14170 29516555

[9] Orsolini L Chiappini S Papanti D De Berardis D Corkery JM Schifano F. The bridge between classical and “synthetic”/chemical psychoses: towards a clinical, psychopathological, and therapeutic perspective. Front Psychiatry. (2019) 10:851. doi: 10.3389/fpsyt.2019.00851 31849723 PMC6896660

[10] Schifano F Napoletano F Chiappini S Guirguis A Corkery JM Bonaccorso S. New/emerging psychoactive substances and associated psychopathological consequences. psychol Med. (2021) 51:30–42. doi: 10.1017/S0033291719001727 31327332

[11] Ricci V Ceci F Di Carlo F Lalli A Ciavoni L Mosca A. Cannabis use disorder and dissociation: a report from a prospective first-episode psychosis study. Drug Alcohol Dependence. (2021) 229:109118. doi: 10.1016/j.drugalcdep.2021.109118 34688166

[12] Ricci V Di Muzio I Ceci F Di Carlo F Mancusi G Piro T. Aberrant salience in cannabis-induced psychosis: a comparative study. Front Psychiatry. (2024) 14:1343884. doi: 10.3389/fpsyt.2023.1343884 38260781 PMC10801803

[13] American Psychiatric Association. Diagnostic and Statistical Manual of Mental Diseases (DSM-IV). 4th ed. Washington, DC: American Psychiatric Publishing (1994).

[14] Curran HV Freeman TP Mokrysz C Lewis DA Morgan CJ Parsons LH. Keep off the grass? Cannabis, cognition and addiction. Nat Rev Neurosci. (2016) 17:293–306. doi: 10.1038/nrn.2016.28 27052382

[15] Mathias S Lubman DI Hides L. Substance-induced psychosis: a diagnostic conundrum. J Clin Psychiatry. (2008) 69:358–67. doi: 10.4088/JCP.v69n0304 18278990

[16] Wolf RC Werler F Schmitgen MM Wolf ND Wittemann M Reith W. Functional correlates of neurological soft signs in heavy cannabis users. Addict Biol. (2023) 28. doi: 10.1111/adb.13270 36825488

[17] Beheshti I. Cocaine destroys gray matter brain cells and accelerates brain aging. Biology. (2023) 12:752. doi: 10.3390/biology12050752 37237564 PMC10215125

[18] Thomas DM Kuhn DM. Attenuated microglial activation mediates tolerance to the neurotoxic effects of methamphetamine. J Neurochemi. (2005) 92:790–7. doi: 10.1111/j.1471-4159.2004.02906.x 15686480

[19] Vilca SJ Margetts AV Höglund L Fleites I Bystrom LL Pollock TA. Microglia contribute to methamphetamine reinforcement and reflect persistent transcriptional and morphological adaptations to the drug. Brain Behav Immun. (2024) 120:339–51. doi: 10.1016/j.bbi.2024.05.038 PMC11269013 38838836

[20] Murnane KS Edinoff AN Cornett EM Kaye AD. Updated perspectives on the neurobiology of substance use disorders using neuroimaging. Subst Abuse Rehabil. (2023) 14:99–111. doi: 10.2147/SAR.S362861 37583934 PMC10424678

[21] Hines LA Freeman TP Gage SH Zammit S Hickman M Cannon M. Association of high-potency cannabis use with mental health and substance use in adolescence. JAMA Psychiatry. (2020) 77:1044–51. doi: 10.1001/jamapsychiatry.2020.1035 PMC7254445 32459328

[22] Liang X Avram MM Gibbs-Dean T Chesney E Oliver D Wang S. Exploring the relationship between frequent cannabis use, belief updating under uncertainty and psychotic-like symptoms. Front Psychiatry. (2024) 15:1309868. doi: 10.3389/fpsyt.2024.1309868 39114739 PMC11304345

[23] Baptiste-Roberts K Hossain M. Socioeconomic disparities and self-reported substance abuse-related problems. Addict Health. (2018) 10:112–22. doi: 10.22122/ahj.v10i2.561 PMC6494986 31069035

[24] De Clérambault GG. Oeuvres psychiatriques . In: . A Cura Di Henri Ey.Presses Universitaires de France, Paris (1942).

[25] Bonhoeffer K. Klinische und anatomische Beiträge zur Kenntnis der Alkoholdelirien. Eur Neurol. (1897) 1:229–51.

[26] De Clérambault GG. Définition de l’Automatisme Mental. In: Oeuvres Psychiatriques. Frénésie éditions, Paris, France (1987). p. 492–4.

[27] De Clérambault GG. Les psychoses hallucinatoires chroniques. In: Oeuvres Psychiatriques;. Frénésie éditions, Paris, France (1987). p. 495–526.

[28] De Clérambault GG. Du Rôle de l’Affectivité dans les Psychoses Hallucinatoires Chroniques. In: Oeuvres Psychiatriques. Frénésie éditions, Paris, France (1987). p. 580–7.

[29] De Clérambault GG. Discussion du Rapport de M. Nayrac sur l’Automatisme Mental au Congrès de Blois. In: Oeuvres Psychiatriques. Frénésie éditions, Paris, France (1987). p. 587–99.

[30] Ey H. Des Idées de Jackson à un Modèle Organo-Dynamique en Psychiatrie. Toulouse, France: Privat (1975).

[31] Fierens C. L’automatisme mental de Clérambault dans le séminaire de Lacan. J Français Psychiatr. (2017) 2:41–8.

[32] Ricci V Maina G Martinotti G. Rethinking mental automatism: de clérambault’s theory in the age of novel psychoactive drugs. Healthcare. (2022) 12:1172. doi: 10.3390/healthcare12121172 PMC11202699 38921287

[33] Lacan J. The Seminar of Jacques Lacan, Book III: The Psychoses (1955-1956). New York: W.W. Norton & Company (1993).

[34] Neumärker KJ. Karl Bonhoeffer. Leipzig, Germany S. Hirzel Verlag (1990).

[35] Bonhoeffer K. Zur frage der klassifikation der symptomatischen psychosen. Berliner Klinische Wochenschrift. (1908) 45:2257–60.

[36] Schwarz J. Biography of K. Bonhoeffer. In: Biographical Archive of Psychiatry (BIAPSY) Biapsy - Biographical Approaches to the Historyof Psychology. Würzburg, Germany: Institute of Psychology, University of Würzburg. (2015). Available at: https://biapsy.de/index.php/en/.

[37] Callieri B. Contributo allo Studio Psicopatologico degli Effetti della Monoetilamide dell’Acido Lisergico. In: Le Psicosi Sperimentali. Feltrinelli, Milan, Italy (1962). p. 139–94.

[38] Cargnello D. Gli Aspetti Psicopatologici della Intossicazione Sperimentale da LSD nei Normali. In: Le Psicosi Sperimentali. Feltrinelli, Milan, Italy (1962). p. 61–138. 13545992

[39] Angrist BM Gershon S. The phenomenology of experimentally induced amphetamine psychosis—preliminary observations. Biol Psychiatry. (1970) 2:95–107. 5459137

[40] Wearne TA Cornish JL. A comparison of methamphetamine-induced psychosis and schizophrenia: A review of positive, negative, and cognitive symptomatology. Front Psychiatry. (2018) 9:491. doi: 10.3389/fpsyt.2018.00491 30364176 PMC6191498

[41] Lecomte T Lang D Potvin S Diotte F Livet A Cimaglia M. SIPD or psychotic disorder with stimulant use. Schizophr Res: Cogn. (2024) 39:100332. doi: 10.1016/j.scog.2024.100332 39430164 PMC11490665

[42] Ahmadkhaniha H Ayazi N Alavi K Najjarzadehgan M Hadi F. The comparison between positive and negative symptoms severity in prolonged methamphetamine-induced psychotic disorder and schizophrenia. Basic Clin Neurosci. (2022) 13:325–33. doi: 10.32598/bcn.2021.2837.1 PMC9706289 36457876

[43] Bleuler E. Die prognose der dementia praecox (Schizophreniegruppe). Allgemeine Z für Psychiatr und Psych.-Gerichtliche Medizin. (1908) 31:436–64.

[44] Schneider K. Psicopatologia Clinica. Rome, Italy: Città Nuova (1983).

[45] Huber G Gross G. The concept of basic symptoms in schizophrenic and schizoaffective psychoses. Recent Prog Med. (1989) 80:646–52. 2697899

[46] Bramness JG Hjorthøj C Niemelä S Taipale H Rognli EB. Discussing the concept of substance-induced psychosis (SIP). Psychol Med. (2024) 54(11):2852–6. doi: 10.1017/S0033291724001442 39252388

[47] Di Petta G Tittarelli D. Le psicosi sintetiche. In: Il Contributo Della Psicopatologia Fenomenologica Italiana Alle Psicosi Indotte da Sostanze. Giovanni Fioriti Editore, Rome, Italy (2016).

[48] Ricci V Maina G Martinotti G. The loss of spatiality and temporality in twilight consciousness: the emergence of exogenous psychosis induced by novel psychoactive substances. Psychopathology. (2024) 57:248–58. doi: 10.1159/000536076 38631303

[49] Martinotti G De Risio L Vannini C Schifano F Pettorruso M Di Giannantonio M. Substance-related exogenous psychosis: a postmodern syndrome. CNS Spectrums. (2021) 26:84–9. doi: 10.1017/S1092852920001479 32580808

[50] Wang LJ Lin SK Chen YC Huang MC Chen TT Ree SC. Differences in clinical features of methamphetamine users with persistent psychosis and patients with schizophrenia. Psychopathology. (2016) 49:108–15. doi: 10.1159/000445065 27071042

[51] Insel TR. Rethinking schizophrenia. Nature. (2010) 468:187–93. doi: 10.1038/nature09552 21068826

[52] Battaglia S Avenanti A Vécsei L Tanaka M. Neurodegeneration in cognitive impairment and mood disorders for experimental, clinical and translational neuropsychiatry. Biomedicines. (2024) 12:574. doi: 10.3390/biomedicines12030574 38540187 PMC10968650

[53] Battaglia S Nazzi C Thayer JF. Genetic differences associated with dopamine and serotonin release mediate fear-induced bradycardia in the human brain. Trans Psychiatry. (2024) 14:24. doi: 10.1038/s41398-024-02737-x PMC10789820 38225222

[54] Gregorio FD Battaglia S. The intricate brain-body interaction in psychiatric and neurological diseases. Adv Clin Exp Med. (2024) 33:321–6. doi: 10.17219/acem/185689 38515256

[55] Hamilton I. Cannabis, psychosis and schizophrenia: unravelling a complex interaction. Addiction. (2017) 112:1653–7. doi: 10.1111/add.13826 28419656

[56] Rognli EB Heiberg IH Jacobsen BK Høye A Bramness JG. Transition from substance-induced psychosis to schizophrenia spectrum disorder or bipolar disorder. Am J Psychiatry. (2023) 180:437–44. doi: 10.1176/appi.ajp.22010076 37132221

[57] Degenhardt L Saha S Lim CC Aguilar-Gaxiola S Al-Hamzawi A Alonso J. The associations between psychotic experiences and substance use and substance use disorders: findings from the World Health Organization World Mental Health surveys. Addiction. (2018) 113:924–34. doi: 10.1111/add.v113.5 PMC5895500 29284197

